# Effects of flow-responsive milking on milking performance of an automatic milking installation

**DOI:** 10.3168/jdsc.2025-0819

**Published:** 2025-07-30

**Authors:** D.J. Reinemann, C.O. Paulrud, Johan ter Weele

**Affiliations:** 1 Department of Biological Systems Engineering, University of Wisconsin–Madison, Madison, WI 53706; 2 DeLaval International, Tumba, Sweden, 147 14

## Abstract

The objective of this study was to compare a quarter-level vacuum-control system that maintains teat-end vacuum during milking to a similar system with conventional vacuum-control technology for which teat-end vacuum is reduced as milk flowrate increases. A study was performed on a pen of cows that had free access to one automatic milking system with conventional vacuum control and another automatic milking system with flow-responsive vacuum. The study ran for 111 d, during which time the pen contained between 120 and 128 cows that had free access to either milking station, producing a dataset with more than 32,000 milkings. The conventional control system maintained the receiver vacuum at 46 kPa, with a resulting vacuum drop at the teat end proportional to milk flowrate, while the flow-responsive treatment maintained a short-milk-tube vacuum at 45 kPa for the entire milking process. The milk removal rate was 12% greater for the flow-responsive system compared with the conventional vacuum system when measured over the milking station occupancy time and when measured by the milking time (first teat cup attached to last teat cup removed). The quarter-level milk removal rate was 16% higher for the flow-responsive treatment. The milk yield per milking compared with the expected milk yield, based on milk production rate and time since last milking, was 102 g higher at the udder level and 22 g higher at the quarter level in the flow-responsive system than in the conventional vacuum-control system, indicating more complete milking.

## Highlights

• Precision control of milking vacuum level according to milk flow rate allows for using higher milking vacuum during the peak flow period.
• Automated premilking stimulation reduces low milk flow early in milking.
• Quarter-level teat cup removal reduces low milk flow late in milking.
• The combination of these control strategies allows for faster milking while reducing teat congestion.

## Graphical Abstract

**Figure:**
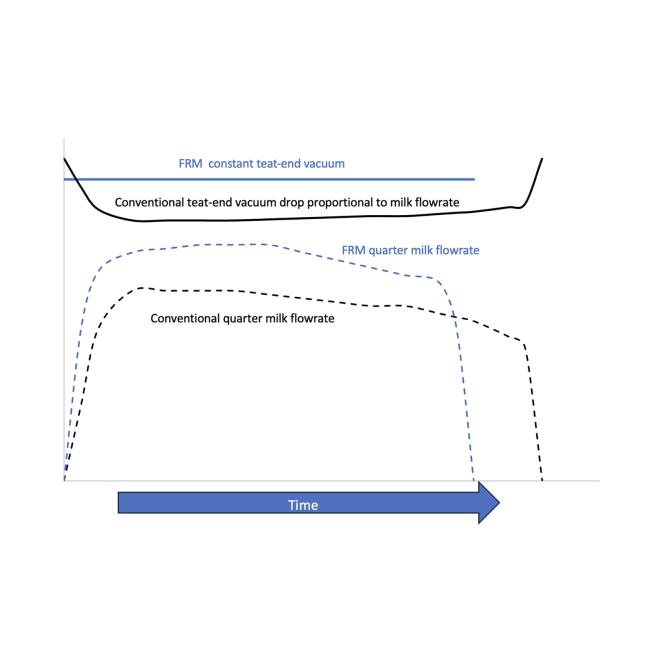
**Summary:** This paper presents the results of a field test of a vacuum-control system, flow-responsive milking (FRM), applied to the DeLaval VMS automatic milking system that maintains teat-end vacuum during the milking process with the aim of reducing milking duration. This FRM system was implemented on an automatic milking installation and resulted in increased milk flow rates at the udder and quarter level, with indications of more complete milking, and demonstrated the potential to improve the capacity of automatic milking installations.

With conventional vacuum control in a milking machine there is an inverse relationship between milk flowrate and teat-end vacuum, with the highest teat-end vacuum occurring at the lowest milk flowrate. Teat congestion is therefore most severe during the low-flow period of milking when the teat-end vacuum level is the highest—approaching the system vacuum (Besier et al., 2016). The regulated milking system vacuum level is normally constant, ranging from 40 kPa in low-level milk lines up to 48 kPa for high-level milk lines, the goal being to account for vacuum drop and achieve teat-end vacuum between 32 and 42 kPa during the peak-flow period of milking as a guideline in the ISO standard for milking machine construction and performance (ISO, 2007). The relationship between milk flowrate and vacuum drop results in claw vacuum approaching the system vacuum during the low-flow period of milking. When quarter-milking technology is applied, as in an automatic milking installation, the teat-end vacuum of each quarter is dependent on the milk flowrate from that quarter rather than the udder total milk flowrate as when conventional clusters are used. DeLaval (Tumba, Sweden) recently introduced a vacuum-control strategy, flow-responsive milking (**FRM**), which maintains teat-end vacuum at a constant level throughout the milking process for automatic milking installations. The objective of this study was to compare milking speed and completeness of milking of a quarter-level vacuum-control system that maintains teat-end vacuum during milking to a similar system with conventional vacuum-control technology for which teat-end vacuum is reduced as milk flowrate increases.

Previous studies of FRM have been done in with conventional cluster milking applications (Stauffer et al., 2020; Reinemann et al., 2021; Singh et al., 2024) and concluded that FRM applied at the cluster level increased peak milk flowrate and average milk flowrate while not having a negative effect on post-milking teat tissue condition. The application of FRM on automatic milking installations is different from the application in conventional or cluster milking systems. In conventional or cluster milking systems FRM applies a low milk-line vacuum during the low-flow period of milking and increases the vacuum to some higher level when udder-level milk flowrate exceeds 2 kg/min. Vacuum drop in the long milk tube results in reduced teat-end vacuum as the milk flowrate increases above these set points. In the automatic milking application the FRM system maintains teat-end vacuum at a constant level for the entire milking process. Our hypothesis was that FRM would increase milk flowrate when compared with the conventional vacuum-control strategy and have a larger effect on milk flowrate than FRM applied in the conventional or cluster application. This is the first peer-reviewed study assessing quarter-level FRM applied in an automatic milking system.

The study farm, in northern Italy, was equipped with DeLaval voluntary milking system (**VMS**) V300 automatic milking systems. One pen of Holstein cows had a free-flow cow traffic system in which cows had free access to 2 VMS, 1 with conventional vacuum-control technology and 1 with FRM. The conventional treatment operated by maintaining constant 46-kPa vacuum in the milk receiver during the entire milking process, resulting in teat-end vacuum of about 39 kPa for a quarter milk flowrate of 1 kg/min. In the FRM system the teat-end vacuum was maintained at 45 kPa during the entire milking process. Individual quarter teat cups were removed when the quarter milk flowrate was less than 0.2 kg/min for both the treatments. A pulsation rate of 60 pulsations/min, pulsator ratio of 72:28, and liner model 999007 (dry test phase durations a = 88 ms, b = 593 ms, c = 67 ms, and d = 200 ms) were the same for both treatments. This study was conducted under University of Wisconsin–Madison Institutional Animal Care and Use protocol ID A005167-R03.

Data were collected for 111 d during which time the pen contained between 120 and 128 cows that had free access to either milking station. Over the entire observation period there were 182 cows that spent some time in the pen and a total of 38,298 milkings. The FRM system averaged 175 milkings/d and the conventional system averaged 170 milkings/d; the overall box occupancy was 17.9 h/d. For purposes of comparing the 2 systems data were filtered to include only cows with at least 20% of their milkings in the alternate treatment and the sample of cows used for this analysis were considered to be quasirandomized to treatment. Data were further filtered to include cows that had at least 6 milkings during the experimental period. These filtering steps reduced the total number of cows used for the analysis to cows in lactations 2 (n = 79), 3 (n = 41), 4 (n = 20), and 5 (n = 7) for a total of 147 cows averaging 166 DIM and a final dataset of 30,205 milkings. The average production level of the study group was 41.9 kg/cow per day with an average milking frequency of 2.8 times/d and average milk yield per milking of 15.2 kg.

For purposes of estimating differences in the completeness of milking an expected milk yield was calculated for each quarter at each milking based on that quarter's milk production rate and time since last milking. The difference between the expected milk yield and the actual milk removed was calculated. Completeness of milking was assessed using the following logic. The milk in the quarter at the time of milking is a combination of the milk left in the quarter at the previous milking and the milk accumulated in the quarter from milk synthesis. If one treatment milked more completely than the other, then the difference between the expected yield and actual yield would indicate a difference in the completeness of milking when one treatment was preceded by the opposite treatment. The effect of treatment on average milk flowrates at the cow level was assessed using a generalized linear model (**GLM**) with cow as a repeated measure. The effect of treatment on average and peak milk flowrates at the quarter level was assessed using a GLM with cow as a repeated measure and quarter nested in cow. The effect of treatment sequence on the difference between expected and actual milk was assessed at the cow level using a GLM with cow as a repeated measure. The effect of treatment sequence on the difference between expected and actual milk was assessed at the quarter level using a GLM with cow as a repeated measure and quarter nested in cow (Table 1).

**Table 1:** Least squares means of outcome variables for milking performance analysis

Level	Conventional	FRM	FRM effect
Cow			
Milk yield per box time (kg/min)	2.38	2.65	+12%
Milk yield per milking time (kg/min)	3.04^a^	3.41^b^	+12%
Actual − expected milk yield (g)	1^a^	103^b^	+102 g
Quarter			
Milk yield per milking time (kg/min)	1.26^a^	1.47^b^	+16%
30-s average maximum milk yield (kg/min)	1.76^a^	2.05^b^	+16%
Actual − expected milk yield (g)	−1^a^	21^b^	+22 g

a,b Differing superscripts within a row indicate differences at the *P* < 0.001 significance level.

The FRM treatment increased the milk removal rate per minute of box time and per minute of milking time (first teat cup on to last teat cup off) at the cow level by 12% (*P* < 0.001). Assuming an average milk yield per milking of 15.2 kg, milking frequency of 2.8 per day, and 20 h of active milking time per day (top 10% of VMS installations in the United States), the application of FRM could increase the number of cows per automatic milking unit from 67 to 75 cows and the milk harvested per box per day from 2,870 to 3,180 kg. The FRM treatment increased the milk removal rate per minute of milking time and the 30-s average peak milk flowrate by 16% (*P* < 0.001). The difference between actual and expected milk yield per milking for the FRM was 102 g higher at the udder level (*P* < 0.001) and 22 g higher at the quarter level (*P* < 0.001) than for the conventional vacuum-control system for milkings that were preceded by the opposite treatment, an indication of more complete milking for the FRM vacuum-control strategy.

We did not assess post-milking teat condition in this study; however, the study farm did not report any concerns and a survey of 14 farms that have implemented FRM on automatic milking systems showed a 1% reduction in incomplete milkings and a 19% reduction in kickoffs during the first week of FRM compared with the prior week using conventional vacuum control. Previous studies using FRM on conventional clusters in which high vacuum was applied during the peak-flow period of milking concluded that teat congestion could be managed by minimizing the low-flow period of milking (Besier and Bruckmaier, 2016). A review by Odorčić et al. (2019) concluded that teat wall thickness was primarily affected by overmilking. A study by Tuor et al. (2023) concluded that milking at a very high vacuum can increase milking efficiency as long as the milk removal rate does not exceed the rate at which milk is delivered by the gland. They further indicated that a vacuum reduction at the start and toward the end of milking is required to prevent teat tissue congestion.

Stauffer et al. (2020) reported that FRM applied at the cluster level produced no change in teat tissue thickness or teat wall diameter between a system using high vacuum during the peak-flow period and reduced vacuum during the low-flow period. Another study of FRM applied at the cluster level reported that the occurrence of rough teat ends was slightly reduced and there were no meaningful differences in the occurrence of teats with blue color, palpable rings, or petechia during the FRM treatment period (Reinemann et al., 2021). Singh et al. (2024) reported that when FRM was applied at the cluster level in combination with adjustment of pulsation during the low-flow period, peak milk flowrates increased and teat condition improved. In contrast to FRM applied at the cluster level, FRM applied at the quarter level with a flow threshold of 200 g/min effectively eliminates the low-flow period at the end of milking so that vacuum reduction is not necessary to prevent teat congestion.

The effect of FRM at the udder level was a 4% increase in the average milk removal rate, from 2.24 kg/min to 2.35 kg/min (*P* < 0.0001), when compared with conventional vacuum control (Reinemann et al., 2021). The effect size of FRM applied at the quarter level in an automatic milking system in this study was larger, with a 12% increase in the milk removal rate from 3.04 to 3.41 kg/min. This increase in milking speed is the result of the combination of (1) higher teat-end vacuum during the peak-flow period, (2) minimizing the low-flow period through automated premilking stimulation and early detachment at the quarter level, and (3) a variable milking interval ensuring that low-fill udders are not milked. This experiment was performed on one farm and the results are generalizable to similar farms using VMS and milking high-producing multiparous cows. Similar improvements in milking speed have been observed for primiparous cows, although controlled studies for primiparous cows and other breeds are recommended for validation.
